# Planning for Sunlight: The Orientation of Buildings

**Published:** 1913-02-15

**Authors:** 


### PLANNING FOR SUNLIGHT.*

### The Orientation of Buildings.

In the case of modern hospitals the questions of site and the location of the various buildings upon it represent important problems. The way in which these questions have been met by architects and hospital constructors in various countries shows by its contradictions the confusion which at present generally prevails. Mr. Atkinson will do well in a second edition, if one is called for, to point out with emphatic clearness that the situation of a site which may be ideal for sunlight purposes might hygienically constitute an infinite danger to the health of the inmates of the buildings erected thereon. Thus, a site well elevated above the surrounding country, despite all the sunlight in the world, if it were exposed to winds blowing over marshes of a malarious nature would be in effect the reverse of a healthy one. Every hospital constructor of any reputation must realise that the primary consideration as regards aspect is to obtain the freest possible access of sunlight to all parts of the site. There is no novelty, therefore, in a book which directs attention solely to planning for sunlight. That such a book may be needed in the United States of America few who are familiar with the ever-increasing height attained by buildings in the city of New York, for instance, can doubt. How New Yorkers dare continuously to disregard the effect in their city of tall buildings overshadowing and shutting out the sunlight from the streets is a problem the solution of which is not apparent. Possibly it may be found in the rush and turmoil which constitutes the business life of certain types of American citizens, whose restless energy leads every year to an increase in the number of relatively young men who break down and disappear in the battle for life.

We are not sure that this book will appeal greatly to British or Continental readers except in the case of those who delight in problems affected by astronomical data and entailing a close study of shadow diagrams and relatively new theories which have yet to be demonstrated as final, or in some cases even as reliable. Still Mr. Atkinson's work is one which those interested in hospital construction may properly add to their library. In the newer hospitals of Germany treatment by sunlight has been elevated to a separate department, with elaborately constructed chambers and not a few appliances. In practice regular treatment of this kind is found to be difficult, if not impossible, owing to the great irregularity and sometimes the infrequency of continuous hours of sunlight during an adequate period of days or weeks or months.

Mr. Atkinson begins with a chapter dealing with the elements of astronomy, which he considers necessary to a clear understanding of the apparent motion of the sun and the variations in the angles of sunlight at different seasons. He also describes the method of the stereographic projection, by which it is claimed the angles of sunlight may easily be obtained for any season of the year and for any latitude. He then treats of the distribution of sunlight upon the exterior of buildings and its admission to their interior, in the course of which treatise he endeavours to apply "the principles of descriptive geometry to the recording of transitory occurrences" which he claims as new. Although he gives some general principles for the planning and placing of buildings, he warns the reader that "each case must be stated as a separate problem, with reference to local conditions, and especially with reference to the latitude of the place." He devotes a whole chapter to the distribution of sunlight in streets as affected by their direction and width and the height of the buildings upon them. His third chapter is devoted to hospitals, in which he discusses "the vexed question of the best orientation for hospital ward pavilions," makes recommendations "at variance with common practice," and gives a plan for "a new type of hospital building." He has therefore planned the "pyramidal type" of ward construction in which each storey is less in area than the one below. He claims for this plan that it fulfils the requirements of modern medical treatment and provides for the classification of patients according to their needs and for the open-air treatment of cases. He places his ordinary pavilions no further apart than is necessary to secure adequate sunlight and air. Adjacent to these pavilions, but not between them, he provides open areas easily accessible to the patients, an idea taken from the Rudolf Virchow Hospital. Each pavilion provides for a subdivision of the patients within itself, and contains two or more open wards of moderate size and a number of smaller wards and single rooms. All the open wards have ridge or monitor ventilation, which prevents one ward being superposed upon another. Ample facilities are provided for open-air treatment on terraces or balconies opening directly from the wards, and also roof balconies or loggias screened in and protected with blinds for patients to sleep in the open air. A day-room is placed in the centre between the main ward pavilions, and ample bathrooms, lavatories, and other service rooms are divided by a corridor from the ward pavilions. He claims for his idea that it solves the problem of the American architect, who must provide covered corridors for climatic reasons between the hospital buildings and secure compactness in plan in the case of large city hospitals. Mr. Atkinson claims for the pyramidal type, of which full plans are given in his book, that it combines the economical advantages of the three-storey type and its adaptability to the subdivision of patients, while it retains the most valuable features of the one-storey type—namely, ridge ventilation. In an Appendix Mr. Atkinson gives in full the building laws of Paris regulating the height of buildings and a synopsis of the regulations of some American cities in this matter.

---

* *The Orientation of Buildings or Planning for Sunlight.* By William Atkinson, Fellow of the Boston Society of Architects. New York: John Wiley and Sons; London: Chapman and Hall, Ltd.

